# A novel computational method enables RNA editome profiling during human hematopoiesis from scRNA-seq data

**DOI:** 10.1038/s41598-023-37325-4

**Published:** 2023-06-26

**Authors:** Yan Wu, Shijie Hao, Xiaojing Xu, Guoyi Dong, Wenjie Ouyang, Chao Liu, Hai-Xi Sun

**Affiliations:** 1 College of Life Sciences, University of Chinese Academy of Sciences, Beijing, 100049 China; 2 BGI-Shenzhen, Shenzhen, 518083 China; 3 BGI-Beijing, Beijing, 102601 China

**Keywords:** Cell biology, Computational biology and bioinformatics

## Abstract

RNA editing is a post-transcriptional modification with a cell-specific manner and important biological implications. Although single-cell RNA-seq (scRNA-seq) is an effective method for studying cellular heterogeneity, it is difficult to detect and study RNA editing events from scRNA-seq data because of the low sequencing coverage. To overcome this, we develop a computational method to systematically identify RNA editing sites of cell types from scRNA-seq data. To demonstrate its effectiveness, we apply it to scRNA-seq data of human hematopoietic stem/progenitor cells (HSPCs) with an annotated lineage differentiation relationship according to previous research and study the impacts of RNA editing on hematopoiesis. The dynamic editing patterns reveal the relevance of RNA editing on different HSPCs. For example, four microRNA (miRNA) target sites on 3ʹ UTR of *EIF2AK2* are edited across all HSPC populations, which may abolish the miRNA-mediated inhibition of *EIF2AK2*. Elevated *EIF2AK2* may thus activate the integrated stress response (ISR) pathway to initiate global translational attenuation as a protective mechanism to maintain cellular homeostasis during HSPCs’ differentiation. Besides, our findings also indicate that RNA editing plays an essential role in the coordination of lineage commitment and self-renewal of hematopoietic stem cells (HSCs). Taken together, we demonstrate the capacity of scRNA-seq data to exploit RNA editing events of cell types, and find that RNA editing may exert multiple modules of regulation in hematopoietic processes.

## Introduction

RNA editing alters the sequence of RNA transcripts dynamically and flexibly during cell development and in a cell type-specific manner^1^. There are two canonical editing types in mammals, A-to-I and C-to-U^2^. Among them, A-to-I is the most common form in animal cells^3^. Most known RNA editing sites located in introns, and 5ʹ/3ʹ untranslated regions (UTRs), especially in the ALU repeat. A small proportion takes place in the coding sequences (CDSs), thus altering the sequence and function of their encoded proteins^4–6^. RNA editing increases the diversity of the transcriptome and expands the ways of regulation^7^. It has implications for various biological processes such as transcriptional stability and localization, interactions with other primary RNA processing steps such as splicing and polyadenylation, and the biogenesis and functions of small RNAs such as miRNAs and long noncoding RNAs^8–10^. Because RNA editing often occurs in the 3ʹ UTRs, in which miRNA binding sites are enriched, it may change the native binding sites or introduce new binding sites, and then affect miRNA-mediated gene silencing^11,12^. Meanwhile, as one of the important post-transcriptional modifications, RNA editing alters base composition at the transcriptional level also exhibits cell-specific features^13,14^.

Hematopoietic stem cells (HSCs) are defined as a group of cells with the ability to self-renew and reconstitute the hematopoietic system^15,16^. They then differentiate into multipotent (produce most blood cell subsets), oligopotent (lymphoid or myeloid restricted), and unipotent hematopoietic progenitor cells (HPCs)^17^. Hematopoietic stem/progenitor cells (HSPCs) require the collaboration of complex pathways including regulation of the cell cycle, apoptosis, and transcription to maintain the internal homeostasis of differentiation and self-renewal^18–24^. More importantly, only HSCs own the ability of both multi-potency and self-renewal. The multi-potency of HSCs is reflected in the differentiation into different hematopoietic progenitor cells and functional blood cells. The self-renewal of HSCs is to generate HSCs themselves rather than through differentiation of other cells. The HSCs differentiate into hematopoietic progenitor cells to maintain the stability of the hematopoietic system by balancing differentiation and self-renewal^25^. As one of the most widespread post-transcriptional modifications, RNA editing also plays an indispensable role and is an important regulator of hematopoietic stem cell maintenance of differentiation function. For example, highly edited *Azin1* in HSPCs led to an amino acid change and then altered *Azin1* protein (AZI) translocation, and finally enhanced AZI binding affinity for DEAD box polypeptide 1. The edited *Azin1* maintains the normal differentiation process of HSCs, whereas loss of *Azin1* RNA editing makes HSCs proliferate^14^. MiRNAs also play a role in the stemness maintenance of hematopoietic stem cells, lineage commitment of hematopoietic progenitor cells, and function of mature effector cells^26–29^. For example, ectopic expression of *miRNA-181* in B-lymphoid cells of murine bone marrow led to an increase in the proportion of B-lineage cells in vivo and in vitro^30,31^. *MiR-150* also played important roles in hematopoiesis. Some studies have provided evidence that *miR-150* blocks the transition from pro-B to pre-B cells during B-cell maturation^32,33^.

The rapid development of next-generation sequencing technologies has promoted in-depth research on RNA editing, and the current use of RNA-seq data for RNA editing site detection tools include Multi-Sampled Method^34^, GIREMI^35^, RDDpred^36^, RED-ML^37^, etc. Since the development of scRNA-seq, most studies focused on gene expression changes in different cell types to explore cellular heterogeneity. Differences in RNA editing events between cell types had been identified in many species. For example in mice, neurons were typically edited at higher levels than glial cells^38,39^. In Drosophila, RNA editing had been studied in multiple neuronal populations, and each neuronal type had unique RNA editing events^40^. In human, it was also found that there were cell type-specific RNA editing sites and cell type-enriched RNA editing sites in glutamatergic neurons, medial ganglionic eminence-derived GABAergic neurons, and oligodendrocytes^41^. These results suggested that RNA editing may give rise to diverse molecular identities of different cell types. Therefore, the exploration of scRNA-seq data cannot be restricted to the expression level. Here, we developed a computational method to detect RNA editing events from scRNA-seq data, and investigated their dynamics and functions in 8 HSPC populations. By integrating aligned reads of all cells of the same cell type, we obtained the pseudo-Bulk RNA-seq of each cell type, which significantly increase the sequencing depth. Meanwhile, as scRNA-seq is strand-specific, we split the aligned reads into forward and reverse strands to improve the accuracy. Using this computational pipeline, we found RNA editing events occurred dynamically during human hematopoiesis, and some sites were co-edited in different cell types. Consistent with previous studies, most of the RNA editing events were located in 3ʹ UTRs, which may change the target sites of miRNAs and then suppress miRNA-mediated gene silencing.

## Results

### A computational pipeline to identify RNA editing sites of cell types from scRNA-seq data

**Figure 1:**
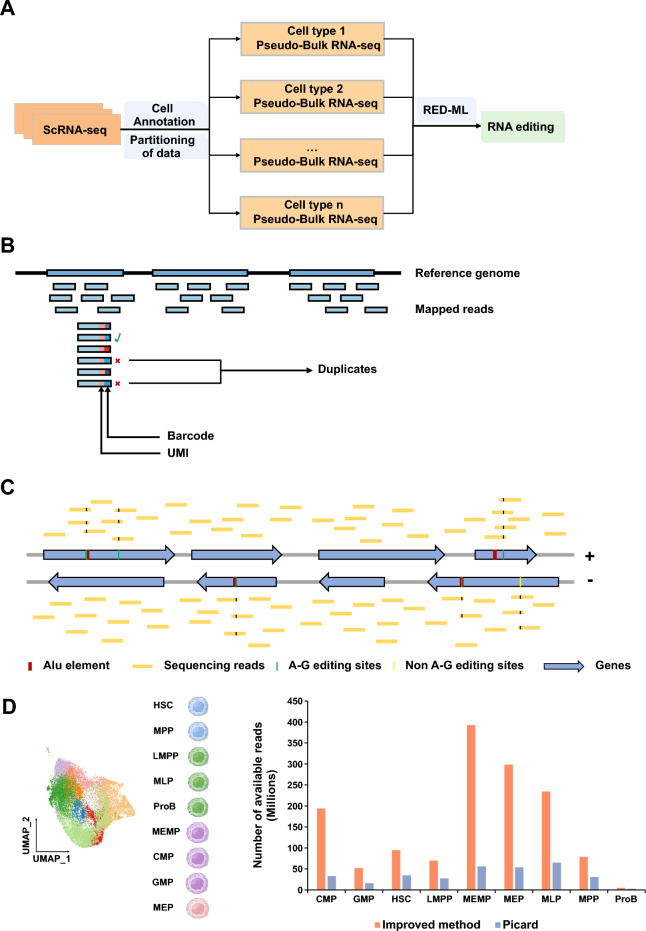
A schematic diagram showing the computational method developed in this study. **(A)** A workflow showing the strategies to identify RNA editing events using scRNA-seq. The cell type annotation information was used to combine the mapped reads of the same cell type in scRNA-seq to obtain pseudo-Bulk RNA-seq for each cell type. **(B)** The novel threshold used to mark duplicates. Only if the aligned reads with the same alignment position, UMI and barcode are defined as duplicates (see details in “Methods” section). **(C)** The reads aligned to the reference genome are divided into reads aligned to the forward strand and reverse strand to distinguish RNA editing sites occurring on the forward/reverse strand genes. The edited sites located in ALU element and with the A-G variation are more likely to be identified as RNA editing sites. **(D)** The scRNA-seq data of hematopoietic stem cells (HSCs), multipotential progenitor cells (MPPs), lymphoid lineage multipotential progenitor cells (LMPPs), multipotential lymphoid lineage progenitor cells (MLPs), megakaryocyte erythroid mast progenitor cells (MEMPs), common myeloid progenitor cells (CMPs), granulocyte monocyte progenitors (GMPs), megakaryocyte erythroid progenitors (MEPs), and B-cell progenitors (ProBs) were used to evaluate improved method and Picard. Bar plot showing the number of available reads using improved method and Picard.

It is a challenge to detect RNA editing events from scRNA-seq data because it has limited sequencing coverages. To overcome this, firstly we integrated the aligned reads of each cell of the same cell type according to the barcode tag in bam files to increase the sequencing coverages, which were merged to a ‘Bulk RNA-seq’ data called pseudo-Bulk RNA-seq data (Fig. 1A) (see details in “Methods” section). Secondly, we improved the method for PCR duplicates removal: only the reads which were aligned to the same genomic coordinate from the same cell with the same unique molecular identifier (UMI) were considered as PCR duplicates (Fig. 1B). Third, as the scRNA-seq data is strand-specific, we split the aligned reads and detect the RNA editing sites of genes transcribed from forward and reverse strands, respectively. In addition, according to the RED-ML method and the features of RNA editing, the sites located in the ALU gene element and the sites mutated as A-G are more likely to be RNA editing sites (Fig. 1C). We applied the improved method to the scRNA-seq data annotated into 8 HSPC populations and found that the improved method could retain more available reads (Fig. 1D).

### Hematopoietic cell lineage and cell type identification

**Figure 2:**
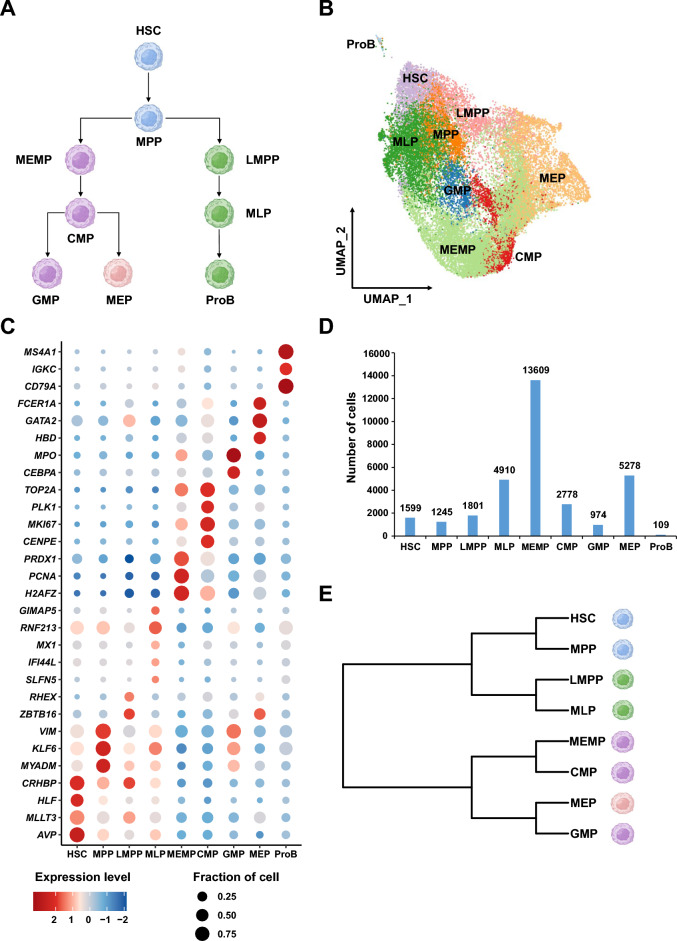
9 HSPCs identified using CB/mPB scRNA-seq data and the exhibition of hematopoietic cell lineage. **(A)** The lineage relationships of HSPCs. According to previous study, HSCs sit at the top of hierarchy. HSCs subsequently differentiate into MPPs. Downstream of MPPs, a strict separation between the myeloid (MEMPs) and lymphoid (LMPPs) branches is the first step in lineage commitment. MEMPs can generate CMPs. CMPs give rise to GMPs and MEPs. LMPPs differentiate into MLPs. MLPs produce ProBs. **(B)** UMAP embedding projection of 32,303 single-cell transcriptomes (query data and reference data). Cell clusters were colored and annotated based on their transcriptional profile identities (see details in Methods). **(C)** Dot plot showing the distribution of expression levels of cell-type-specific marker genes across all 9 cell types (see details in “Methods” section). **(D)** Bar plots showing the total number of detected cells in each cell type. **(E)** Hierarchical clustering is consistent with the hematopoietic cell lineage in previously studied of 8 cell types by transcriptional profiles.

HSPCs is important for maintaining the integrity of the hematopoietic system. To evaluate our method, we used the scRNA-seq data of human HSPCs with defined cell types and lineage relationships according to previous research (Fig. 2A,B). To investigate the conversed RNA editing events of human HSPCs, we collected cord blood (CB) and mobilized peripheral blood (mPB) CD34^+^ cells and obtained their single-cell transcriptomes. Mobilized peripheral blood (mPB) and umbilical cord blood (CB) are two of the current sources of HSPCs. Researches show that CD34^+^ cells derived from CB have stronger regeneration capacities than that from mPB. These samples included 6 CB samples without in vitro cultivation, 6 CB samples with 48-h in vitro cultivation, 3 mPB samples without in vitro cultivation and 3 mPB samples with 48-h in vitro cultivation (Supplemental Table 1, Supplemental Fig. S1A). We can find the conversed RNA editing sites in these different samples and cell culture states. We used a previous dataset with accession code CNP0000978^42^ as reference to identify cell types in our data (Fig. 2B). MLP, MEMP, CMP, GMP, MEP and ProB were annotated in the query datasets based on the expression of marker genes reported in other research (Supplemental Fig. S1B)^42^. Then by integrating the reference and query datasets, we obtained 32,303 cells with specific gene expression profiles (Fig. 2C), including 1599 hematopoietic stem cells (HSCs), 1245 multipotential progenitor cells (MPPs), 1801 lymphoid lineage multipotential progenitor cells (LMPPs), 4910 multipotential lymphoid lineage progenitor cells (MLPs), 13,609 megakaryocyte erythroid mast progenitor cells (MEMPs), 2778 common myeloid progenitor cells (CMPs), 974 granulocyte monocyte progenitors (GMPs), 5278 megakaryocyte erythroid progenitors (MEPs), and 109 B-cell progenitors (ProBs) (Fig. 2D; Supplemental Table 2). As account for only 0.34% (109 of 32,303) of our data, we removed ProBs in further analysis. After generating the pseudo-Bulk RNA-seq data of these cell types, we reconstructed their lineage relationships using unsupervised hierarchical clustering (Fig. 2E), which is consistent with the classical model of lineage determination in human hematopoietic hierarchy (Fig. 2A)^42–44^. HSC and MPP were found to be clustered together. Myeloid and lymphoid hematopoietic cell populations showed close clustering distinctions, respectively. Taken together, these results demonstrated that our scRNA-seq data exhibited the transcriptome features of human HSPCs.

### Dynamic RNA editing events in hematopoietic cell populations

**Figure 3:**
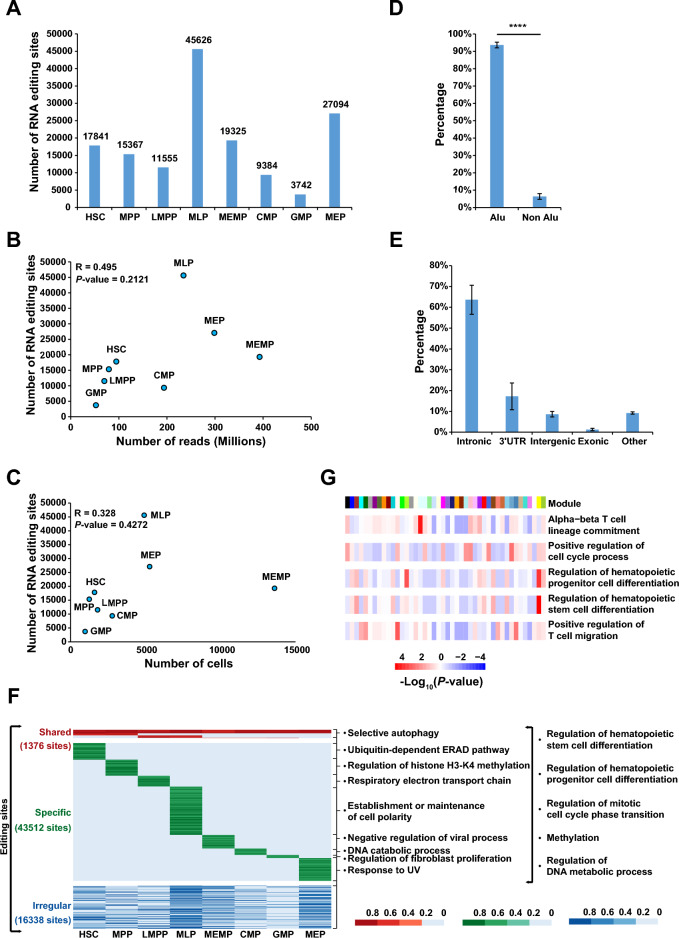
The evaluation of RNA editing sites by distribution in different cell types and genomic elements and their dynamic changes during hematopoiesis. **(A)** The number of RNA editing sites in each HSPC population. **(B)** Pearson correlation coefficient between the total reads count and the number of editing sites in each HSPC population. **(C)** Pearson correlation coefficient between the cell number and the number of editing sites in each HSPC population. The number of RNA editing sites were independent of cell number and sequencing depth. **(D)** Bar plot showing the proportion of editing sites within and outside Alus in HSPC populations. Most RNA editing sites located in Alu genomics element. Data present mean ± s.e.m. *P*-value was calculated by T-Test. ****P < 0.0001. **(E)** Bar plot showing the distribution of editing sites across different genomic elements in HSPC populations. Data present mean ± s.e.m. **(F)** A heatmap showing confidence scores (P_edit) calculated by RED-ML of all editing sites. The color indicates P_edit (from 0 to 1) for a given site (row) in a population (column). The P_edit equal to 0 means this site is not edited in this population whose coverage is greater than 30×. The right listed the most significant enriched GO pathways in each pattern and the common pathways in all patterns. These GO terms were analysed with genes of these RNA editing sites annotated by ANNOVAR. **(G)** A heatmap showing the most significant enriched GO pathways in each module related to hematopoiesis. The genes annotated by ANNOVAR were used to do GO analysis.

Next we used RED-ML^37^ to detect RNA editing sites using the pseudo-Bulk RNA-seq data of each cell type. To reduce false positives, we only retained RNA editing sites with sequencing depths greater than 30×. Then a total of 17,841, 15,367, 11,555, 45,626, 19,325, 9,384, 3,742 and 27,094 editing sites in the eight hematopoietic cell populations after filtering (Fig. 3A), and 98,549 unique RNA editing sites among eight hematopoietic cell populations. The investigation of the distance between two adjacent editing sites revealed that global RNA editing density was stable across the eight hematopoietic populations (Supplemental Fig. S2A). There was no significant correlation between the total number of non-redundant reads, the number of cells, and the number of identified RNA editing sites, indicating that the differences in RNA editing events across HSPCs were not due to technical issues (Fig. 3B,C). In addition, consistent with previous reports^6^, most of the RNA editing sites (94%) were located in the ALU element (Fig. 3D, Supplemental Fig. S2B). To further characterize the RNA editing sites in HSPCs, we investigated the distribution of RNA editing sites in different functional regions of the genome. Most of the RNA editing sites were located in introns (64%) and 3ʹ UTR (17%), and only 1% of the sites were in the coding region (Fig. 3E, Supplemental Fig. S2C), which is in agreement with a previous study^14^.

As RNA editing events are dynamic between cell types (Fig. 3A), to build a comprehensive RNA editing landscape of the eight HSPC populations, we collected RNA editing sites whose read coverage was > 30× in all cell types. A fraction of editing sites (termed shared) was edited in all 8 cell types or specifically edited in lymphoid or myeloid or HSC/MPP hematopoietic cells, while another fraction of editing sites (termed specific) was specifically edited in one cell type (Fig. 3F, Supplemental Tables 3, 4). We then investigated their biological functions and found that RNA editing sites with the above patterns were enriched in different pathways (Fig. 3F). RNA editing may involve in embryonic erythropoiesis^45^ and adult HSPC differentiation^46,47^. We found the shared RNA editing sites and specific RNA editing sites both enriched in the pathway relevant with regulation of hematopoietic stem/progenitor cells differentiation and cell cycle (Fig. 3F), which has been proved in several previous studies that cell cycle may have influence on HSPCs differentiation^20,48^. Those results suggest that RNA editing may regulate the biological processes of HSPCs in multiple ways. In previous studies, it was found that different RNA editing sites may have co-editing modules^14^. RNA editing sites within the same module are highly correlated. Consistent with these studies, we also identified co-editing modules among different editing sites (Fig. S3A). And different modules may play a role in different cell types (Fig. S3B). The functions of these modules were significantly enriched in the regulation of hematopoietic stem cell differentiation, lineage commitment, stem cell population maintenance, transcription initiation, protein assembly and other HSPC-related biological processes (Fig. 3G, Supplemental Fig. S3C). Therefore, this result suggested that these co-edited modules may have biological functions rather than random events. Together, these observations suggested that RNA editing might play specific roles in different cell types, and might be key to lineage differentiation and self-proliferation during hematopoiesis. Some co-edited modules that occurred in the same biological process may serve as another way for regulating human hematopoiesis.

### Possible functions of RNA editing on the maintenance of cell homeostasis during differentiation of HSPCs

**Figure 4:**
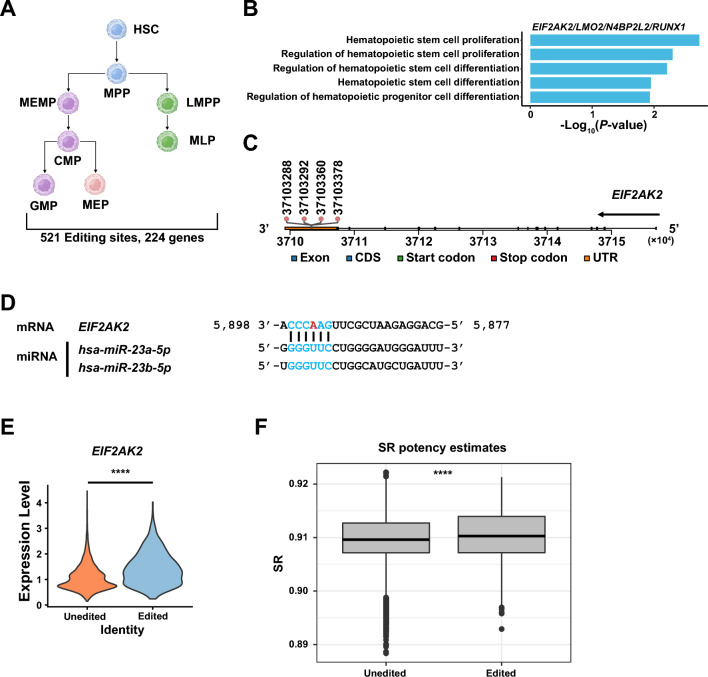
RNA editing may have effects on cell homeostasis during hematopoietic cell differentiation in all HSPCs. **(A)** A summary of the number of editing sites and genes annotated by ANNOVAR shared across 8 HSPC populations. **(B)** The GO result related with HSPCs proliferation and differentiation of shared genes with RNA editing sites located in 3ʹ UTR or exon coding region. **(C)** The track of *EIF2AK2* shows the loci of RNA editing sites located in the 3ʹ UTR. **(D)** Predicted site of *miR-23a* and *miR-23b* in the *EIF2AK2* 3ʹ UTR (red: nucleotides edited, blue: binding site). **(E)** Violin plot showing the expression level of *EIF2AK2* in cells with and without *EIF2AK2* editing sites. *P-*value was calculated by Wilcoxon Rank Sum test. ****P < 0.0001 **(F)** Box plot shows the signaling entropy rate which indicates the differentiation potency of cells in cells without and with *EIF2AK2* editing sites. *P-*value was calculated by T-Test. ****P < 0.0001.

To further explore the global functions of RNA editing on HSPCs, we first analyzed the 521 editing sites shared by all cell types, which were distributed in 224 genes (Fig. 4A, Supplemental Table 5). To explore the role of RNA editing sites in hematopoietic processes, GO enrichment analysis was performed on 224 genes. GO enrichment analysis revealed that *EIF2AK2*, *LMO2*, *N4BP2L2*, and *RUNX1* were involved in regulating the differentiation of hematopoietic stem/progenitor cells and the RNA editing sites of those genes located in 3ʹ UTR and exon which may influence the miRNA binding sites and protein translation. (Fig. 4B, Supplemental Table 6). Among them, *EIF2AK2* was reported to regulate responses to stress during hematopoiesis^49^. In our data we identified 37,103,288, 37,103,292, 37,103,360, 37,103,378 four RNA-editing sites in *EIF2AK2* 3ʹ UTR, which may affect miRNA binding (Fig. 4B,C). Prediction of miRNA targeting sites using TargetScanHuman^50^ revealed that the binding sites of *miR-23a/miR-23b* in *EIF2AK2* 3ʹ UTR were altered due to RNA editing (Fig. 4D, Supplemental Fig. S4A). As *miR-23a*/*miR-23b* were expressed in CD34^+^ cells^51^, we hypothesized that RNA editing may prevent the binding of *miR-23a/miR-23b* to *EIF2AK2,* thereby derepress *EIF2AK2*. Then we tested this hypothesis and found that as expected, for cells expressing *EIF2AK2*, the overall expression levels of *EIF2AK2* were higher in the cells when *EIF2AK2* was edited (log_2_foldchange: 0.56; *P-*value < 0.0001) (Fig. 4E). And we found that *EIF2AK2* editing occurred only in a small fraction of cells (3.11%) (Supplemental Fig. S4B), which was in agreement with a previous study^52^.

Cell pool integrity is maintained in the blood system by clearing damaged cells. But in order to ensure the longevity of the cells^53,54^, it is also necessary to ensure that the cells can survive the lower-level stress that often occurs^49^. Many stressors congregate on the integrated stress response (ISR) pathway, whose function is to balance the pressure signals that activate the cell death pathway in order to protect the cells to restore the cell balance^55^. PKR encoded by *EIF2AK2* is one of the four stress-inducible kinases that maintain the survival-death equilibrium^49^. The interaction between PKR and the other three kinases (HRI, PERK, GCN2) affects the phosphorylation of eIF2α^56^, leading to the attenuation of global translation initiation, which saves amino acid synthesis for essential cellular function, reduces the load of chaperones, and lowers the metabolic demand associated with protein synthesis^57^. Along with the up-regulation of *EIF2AK2*, We found the expression levels of *EIF2AK1*, *EIF2AK3* and *EIF2AK4* (encoding HRI, PERK and GCN2, respectively) were all higher in cells expressing edited *EIF2AK2* compared to those expressing unedited *EIF2AK2* (Supplemental Fig. S4C), implying that RNA editing of *EIF2AK2* may affect the maintenance of cell homeostasis. In addition, we compared the expression levels of *EIF2AK2* in eight cell types carrying *EIF2AK2* edited sites with non-edited cells. We found that the expression levels of *EIF2AK2* in cells with edited sites were higher than those in cells without edited except GMP with no significant difference (Supplemental Fig. S5A). Meanwhile, previous studies found that human HSCs are sensitive to the interference of cell homeostasis. The induction of reactive oxygen species (ROS) and accumulation of DNA damage increase the possibility of apoptosis of HSCs compared to downstream progenitor cells^58,59^. A study has found high integrated stress response activity in HSC/MPPs compared to progenitors which have lower differentiation potential^49^. Therefore, we hypothesized that there is a relationship between the activity of ISR pathway and differentiation potency. To test this hypothesis, we used SCENT^60^ to explore the effect of RNA editing on the differentiation potential of cells. The result also revealed a higher differentiation potential in *EIF2AK2* edited cells (Fig. 4F). High differentiation potential was also shown in cells edited with EIF2AK2 among the 8 HSPC populations with significant differences (Supplemental Fig. S5B). Taken together, based on the above analysis, the RNA editing events in *EIF2AK2* may play an important role in maintaining cell homeostasis. Besides, we speculated that *EIF2AK2* may undergo RNA editing in cells with higher differentiation potential which may more sensitive to interference.

### Impact of RNA editing on the lineage commitment of HSPCs and self-renewal of HSCs

**Figure 5:**
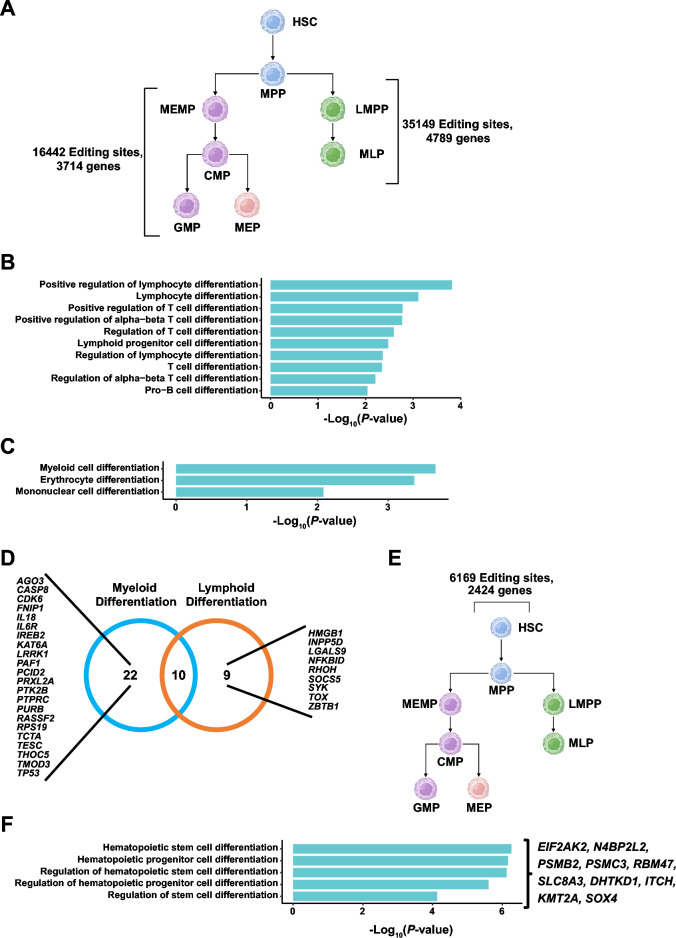
RNA editing might influence lineage commitment and HSCs differentiation and self-renewal. **(A)** A summary of the number of lymphoid-specific and myeloid-specific editing sites and genes annotated by ANNOVAR. **(B)** The enrichment analysis of genes with lymphoid-specific editing sites involved in the differentiation of lymphoid cells. **(C)** The enrichment analysis of myeloid-specific edited genes involved in the differentiation of myeloid cells. **(D)** Venn plot showing the genes edited in lymphoid and myeloid cell lines. The editing sites of those genes are all located in 3ʹ UTR or exon coding region and involved in the differentiation of lymphoid cells, myeloid cells, respectively. **(E)** A summary of the number of HSC-specific editing sites and genes annotated by ANNOVAR. **(F)** The enrichment analysis of HSC-specific edited genes involved in hematopoietic stem cell differentiation. And genes involved in those pathways with editing sites are located in 3ʹ UTR or exon coding region.

Then we investigate the differences in RNA editing between myeloid and lymphoid HSPCs. We obtained 16,442 lymphoid-specific RNA editing sites distributed in 3714 genes, and 35,149 myeloid-specific RNA editing sites distributed in 4789 genes, respectively (Fig. 5A). GO analysis of genes with lymphoid lineage-specific RNA editing sites revealed a strong enrichment involved in differentiation of lymphocytes, differentiation of T cells, and differentiation of B-cell progenitors (Fig. 5B, Supplemental Table 6). Whereas genes in myeloid lineage-specific editing sites are highly involved in the differentiation of myeloid cells, erythroid cells, and monocytes (Fig. 5C, Supplemental Table 6). All GO terms are provided in supplementary materials. Thus, RNA editing may regulate lineage differentiation of lymphoid and myeloid cells, consistent with a published paper showing that RNA editing may contribute to cell fate decision in the hematopoietic system^14^.

To further explore the function of genes involved in myeloid and lymphoid HSPCs differentiation pathways, we focused on RNA editing sites located in the 3ʹ UTR and coding regions which may influence the post-translational modification and the function of proteins. We found that 19 and 32 genes were in the pathways regulating lymphoid HSPCs differentiation and myeloid HSPCs differentiation, respectively. And the RNA editing sites of those genes all occurred in the 3ʹ UTR and exon coding regions (Fig. 5D). Many of these genes play important functions in the hematopoietic system. For example, Caspase 8 encoded by *CASP8* is a cysteine protease and a key mediator of apoptosis^61^. Recently, however, many studies have introduced some new aspects to it, citing their significance in cell development and differentiation. The study by Rebe et al. has proved that caspase 8 activity is required for the differentiation of peripheral blood monocytes into macrophages in myeloid cell lines^62^. Meanwhile, *ZBTB1* regulates the development of lymphoid cell lines and myeloid cell lines. Siggs, Owen et al. have shown that a chemically induced mouse with *Zbtb1* mutation has a complete and cell-intrinsic T cell deficiency. Besides this, other lymphoid cell lines were also partially impaired. The study shows that *ZBTB1* may act as an important transcriptional regulator determining T cell development and lymphopoiesis^63^.

HSCs are the stem cells that give rise to other hematopoietic cells. We find 6169 sites distributed on 2424 genes specifically edited in HSCs (Fig. 5E). GO Enrichment analysis revealed that genes with RNA editing sites were involved in hematopoietic stem cell differentiation and hematopoietic progenitor cell differentiation, and the RNA editing sites of some genes are located in 3ʹ UTR or exon coding region (Fig. 5F, Supplemental Table 6), such as *EIF2AK2*, *N4BP2L2*. According to the GenCards database (https://www.genecards.org/), *N4BP2L2* may be involved in the positive regulation of HSC proliferation and negative regulation of HSC differentiation (Table 1).

**Table 1:** The function of *N4BP2L2* from GeneCards database.

GO ID	Qualified GO term	Evidence	PubMed IDs
GO:1902035	Involved in positive regulation of hematopoietic stem cell proliferation	IMP	19506020
GO:1902037	Involved in negative regulation of hematopoietic stem cell differentiation	IMP	19506020

Our results showed that RNA editing occurred in genes important for regulating lineage commitment, cell differentiation, and self-renewal during hematopoiesis, suggesting an important role for RNA editing in the hematopoietic process.

## Discussion

Differing from previous analysis of scRNA-seq mainly focusing on the gene expression levels, here we provided a novel computational method to study RNA editing using scRNA-seq, and applied it to interpreting hematopoietic differentiation and HSC self-renewal. To perform RNA editing analysis in scRNA-seq data, we made the following three improvements: (i) using cell annotation information to obtain pseudo-Bulk RNA-seq of different cell types; (ii) optimizing the marking duplicates method to increase the sequencing depth; and (iii) using strand information to detect RNA editing sites. Compared with other studies, the distribution of RNA editing sites across different genomic elements, and the proportion of RNA editing sites within ALU elements were consistent with previous research reports^14^.

We then explore the shared RNA editing sites and specific RNA editing sites among different HSPC populations and found that RNA editing is dynamic between different cell types during HSPCs differentiation and self-renewal, and has specific editing events for different cell types. However, our study did not further resolve the underlying mechanisms conferring to this dynamic change. It seems that RNA editing may serve as a molecular marker for different cell types and have implications for the performance of specific functions of different cell types. Since ADAR enzymes catalyze RNA editing events, there may be some differences in the expression of ADAR enzymes in different cell types, all of which need to be investigated in detail by additional work. Moreover, our study found that *EIF2AK2* with the shared RNA editing sites in all HSPC cell populations may have an impact on maintaining self-homeostasis during HSPCs differentiation through ISR pathway. Though a study has found that ISR activation in HSCs/MPPs is more active than in hematopoietic progenitor cells^49^, our findings may indicate that the ISR pathway not only safeguards HSCs/MPPs but also affects all HSPCs with high differentiation potential. This implies that HSPCs with higher differentiation potential may be more sensitive to external stimulus stress. They perform specific regulation of their own cellular homeostasis through RNA editing to maintain the integrity of the hematopoietic system. Meanwhile, we found lymphoid-specific and myeloid-specific RNA editing sites may involve in lineage commitment and HSPCs differentiation. According to a previous study, RNA editing may influence lineage commitment during hematopoiesis. They found the frequency of RNA editing alters at the branch point of HSPCs differentiation^14^. Our results indicate that not only changes in editing frequency, but also specific RNA editing sites occur in different lineages during differentiated development. This may suggest that RNA editing sites occur at genes critical to lineage commitment. Besides shared sites, we found specific editing sites in HSCs may have functions on differentiation and self-renew. The capacity to self-renew and to differentiate into other hematopoietic cells are important features of HSCs^15^. This also suggests that RNA editing may play an important role in maintaining the balance of self-renewal and differentiation of HSCs. However, those results still require more experiments to verify the role of RNA editing for HSPCs.

More broadly, our approach validates the feasibility and usability of RNA editing event studies using high-throughput scRNA-seq. And we showed the overall differences RNA editing of the HSPCs and the possible function. In summary, these efforts confirm the great potential and value of scRNA-seq for the study of biological process mechanisms. Though future studies will be required to confirm and clarify the role of RNA editing.

## Methods

### Accession numbers

The reference dataset (aligned reads) was downloaded from CNSA (https://db.cngb.org/cnsa/) of CNGBdb with accession code CNP0000978. Both the reference and query dataset were CD34^+^ cells obtained from human CB and mPB, and the method of cell cultivation, scRNA-seq libraries and pre-processing were as previously described^42^. The raw sequencing data of query dataset generated in this study was deposited in the CNGB Sequence Archive (CNSA; https://db.cngb.org/cnsa/) of China National GeneBank DataBase (CNGBdb) with accession code CNP0003367.

### Ethics statement

This study was performed with the approval of the Institutional Review Board of BGI (BGI-IRB 16089-T3 and BGI-IRB 22090). All methods were performed in accordance with relevant quidelines and regulations.

### Enrichment of CD34^+^ cells from human CB and mPB samples

Human CB and mPB samples were obtained from healthy donors with informed consent. We got mononuclear cells (MNCs) using centrifugation on Lymphoprep medium. CD34 Microbead kits and LS columns using MACS magnet technology (Miltenyi) were used for MNC enrichment for CD34^+^ cell selection. Downstream experiments were conducted on CD34^+^ cells after sorting.

### Cell culture in vitro and scRNA-seq library

The fresh CD34^+^ cells were applied to cell culture in vitro or to single-cell RNA-seq (scRNA-seq). CD34^+^ cells were resuspended in SCGM medium (Cellgenix) using the following recombinant hematopoietic cytokines: recombinant human stem cell factor (rhSCF) 100 ng/ml, recombinant human thrombopoietin (rhTPO) 100 ng/ml, recombinant human fms-related tyrosine kinase-3 ligand (rhFlt3-L) 100 ng/ml and cultured in 24-well tissue culture plates at 37 °C in an atmosphere of 5% CO_2_ for 48 h (Thermo Fisher). The DNBelab C4 platform was used to perform scRNA-seq. Single-cell suspensions were used for droplet generation, demulsification, microbead collection, reverse transcription, and cDNA amplification to generate barcode libraries. The manufacturer’s protocol was used to construct indexed libraries. QubitTM ssDNA Assay Kit (Thermo Fisher Scientific; #Q10212) was used to quantify the sequencing libraries. DNA nanoballs (DNBs) are loaded into a pattern nanoarray and sequenced at ultra-high throughput with the following read lengths used by the DIPSEQ T1 sequencer: 30 bp for read 1, inclusive of 10 bp cell barcode 1, 10 bp cell barcode 2 and 10 bp unique molecular identifier (UMI), 100 bp of transcript sequence for read 2 and 10 bp for sample index.

### Mapping and annotating query datasets

To identify HSPC populations in CD34^+^ cells, we used “Mapping and annotating query datasets” method of Seurat package (v. 4.1.0) in R (v.4.0.5)^64–67^. This approach allows the comparison of the similarity of cells in the reference and target datasets, resulting in cell annotation of the target dataset with unknown cell types. The reference data set was obtained from CNGBdb with accession code CNP0000978^42^. Then the query dataset was mapped to the reference data for annotating cell types. First, we imported the final cell-gene expression matrix of the query dataset into the Seurat package to create a Seurat objects. Cells with fewer than 200 detected genes and for which the total mitochondrial gene expression exceeded 5% were removed. Besides, we used the IQR Method of Outlier Detection to remove cells with outlier gene number. Genes expressed in fewer than three cells were also removed.

Downstream analyses were also performed using Seurat package (v. 4.1.0). Normalizing SeuratObject used the NormalizeData function and the ScaleData function. The following functions were run together when mapping the query dataset to the reference dataset: FindIntegrationAnchors, TransferData, IntegrateEmbeddings, ProjectUMAP. The FindAllMarkers function was then used to find the cell type-specific genes. Differential expression analysis was performed based on the Wilcoxon Rank Sum test. Finally, the merge function of Seurat was used to merge the query dataset and reference dataset. All methods use default parameters unless otherwise specified.

The AverageExpression function of Seurat was used to calculate the average expression of 8 HSPC cell types. And then we used hclust function to perform the hierarchical clustering.

### Splitting aligned reads based on cell type

To get pseudo-Bulk RNA-seq of 8 cell types, we got the barcode and sample information for each cell of the 8 cell types from SeuratObject. Based on the CB (Cell Barcode) tag in the bam file we can determine the cell from which the reads originated, so we can use the cell barcode of each cell type to obtain aligned reads for each cell barcode. And then, we integrated multiple cells aligned reads of each cell type to obtain a pseudo-Bulk RNA-seq for each cell type. The code of this method is available on Github (https://github.com/Genki-YAN/Cell2Editing).

### Marking duplicates and obtaining strand-specific reads

Considering the difference between scRNA-seq data and Bulk RNA-seq data, we designed the procedure of marking duplicates: taking the aligned position, UMI barcode, and cell barcode of reads into account for the definition of duplicates. Only the reads from the same cell with the same unique molecular identifier (UMI) were considered as PCR duplicates. And we retained the read with the highest base quality. The code of this process is available on Github (https://github.com/Genki-YAN/Cell2Editing).

We split strand-specific aligned reads based on flag 16 (read reverse chain) in bam file using Samtools^68^ with the following commands: samtools view -b -f 16; samtools view -b -F 16.

We compared the number of available reads detected by the improved method with the Picard (“Picard Toolkit.” 2019. Broad Institute, GitHub Repository. https://broadinstitute.github.io/picard/; Broad Institute) method. We counted and compared the non-duplicates reads in the bam files processed by the two methods. The tool locates and marks duplicates in BAM or SAM files, where repeat reads are defined as originating from a single DNA fragment. Duplication may occur during sample preparation, such as library construction using PCR. If the 5’ position, strand and base alignment are the same, Picard compared base alignment quality to mark duplicate reads and available reads.

### Detecting RNA editing sites

After the above pre-process of data, we used the RED-ML (https://github.com/BGIHENG/RED-ML)^37^ with the default parameters to detect the RNA editing sites in the pseudo-Bulk RNA-seq using the GRCh38 genome and GRCh38 SNP database (https://ftp.ncbi.nih.gov/snp/organisms/human_9606_b151_GRCh38p7/VCF/All_20180418.vcf.gz), and removed RNA editing sites with coverage less than 30× to facilitate subsequent analysis. When we investigated cell type-specific RNA editing sites, we used the mpileup function of Samtools for investigating site sequencing coverage, keeping sites with coverage greater than 30× with the following commands: samtools mpileup -B -f -s - output-QNAME -min-MQ 20 -min-BQ 20 -excl-flags DUP.

### Annotation of RNA editing sites

RNA editing sites were annotated utilizing ANNOVAR (https://annovar.openbioinformatics.org/en/latest/)^69^ table_annovar.pl with the reference genome of GRCh38. We used the following commands: -remove -protocol refGene, phastConsElements20way, wgRna, cytoBand -operation g,r,r,r -nastring . -csvout -polish. Based on the strand of the aligned reads, we only retain the RNA editing sites with genes transcribed from the same strand.

### Correlation module analysis of RNA editing sites

To identify the RNA editing sites that were co-edited in different cell types, the co-editing modules of filtered RNA editing sites in 8 cell types were analyzed using WGCNA package (v. 1.70.3)^70,71^. The WGCNAR package was used to estimate the best soft thresholding power for the co-editing module analysis. The minimum power 14, which reached the R2 cut-off of 0.8 for topology model fit, was determined to be the optimal value. The adjacency with the optimal soft-thresholding power estimated above was calculated, and the adjacency was transformed into a topological overlap matrix to calculate the corresponding dissimilarity, and identified 44 co-editing modules with minModuleSize = 30. Then we annotated the RNA editing sites in each module and perform GO enrichment analysis using clusterProfiler R package (v.4.0.5)^72,73^.

### Identification of cells with editing sites

Since RED-ML is a method designed for RNA editing sites by Bulk RNA-seq, and there is no existing method to detect RNA editing sites in individual cells, we used the mpileup function of Samtools to find the aligned reads with RNA editing sites. The cells with at least 1 read supporting RNA editing events were defined as edited cells. Samtools mpileup was performed using the following parameters: samtools mpileup -B -f -s -output-QNAME -min-MQ 20 -min-BQ 20 -excl-flags DUP. To calculate the proportion of cells with edited *EIF2AK2*, we removed cells that did not express *EIF2AK2*.

### Gene expression and functional analysis of RNA editing sites

The expression of genes with RNA editing sites in cells was quantified using ScaleData function of Seurat. To explore the function of editing sites with different editing patterns, we performed GO enrichment analysis using clusterProfiler package (v.4.0.5)^72,73^.

### Prediction of miRNA target sites

TargetScanHuman (https://www.targetscan.org/vert_80/)^50^ was used to predict miRNAs that may target and bind to candidate genes. Then we compared the binding region with the RNA editing sites to determine whether RNA editing sites are located in the miRNA binding region. The reference file of UTR sequence was downloaded from TargetScanHuman website. The version of RNA sequence used was GRCh38.

### Estimating differentiation potency of single cells

To demonstrate that RNA editing may be correlated with differentiation potency, we first removed cells that do not express genes with the RNA editing, and then we calculated the differentiation potency of cells using SCENT (https://github.com/aet21/SCENT)^60^ with the default parameters. The *P-*value was calculated using T-Test.

## Supplementary Information

Supplementary Figure S1.

Supplementary Legends.

Supplementary Figure S2.

Supplementary Figure S3.

Supplementary Figure S4.

Supplementary Figure S5.

Supplementary Tables.

Supplementary Table 3.

Supplementary Table 4.

Supplementary Table 5.

Supplementary Table 6.

## Data Availability

The datasets generated and analysed during the current study are not publicly available due to human genetic resources management and the data in a published article entitled “Stemness-related genes revealed by single-cell profiling of naïve and stimulated human CD34+ cells from CB and mPB” (10.1002/ctm2.1175) being controlled but are available from the corresponding author on reasonable request.
