# Supplementary material for: A novel computational method enables RNA editome profiling during human hematopoiesis from scRNA-seq data
Source: Sci Rep. 2023 Jun 26;13:10335. doi: 10.1038/s41598-023-37325-4 (PMC10293275; doi:10.1038/s41598-023-37325-4)
Supplement: Supplementary file 1 — Supplementary Figure S1. [file 41598_2023_37325_MOESM1_ESM.pdf]

Figure S1

A

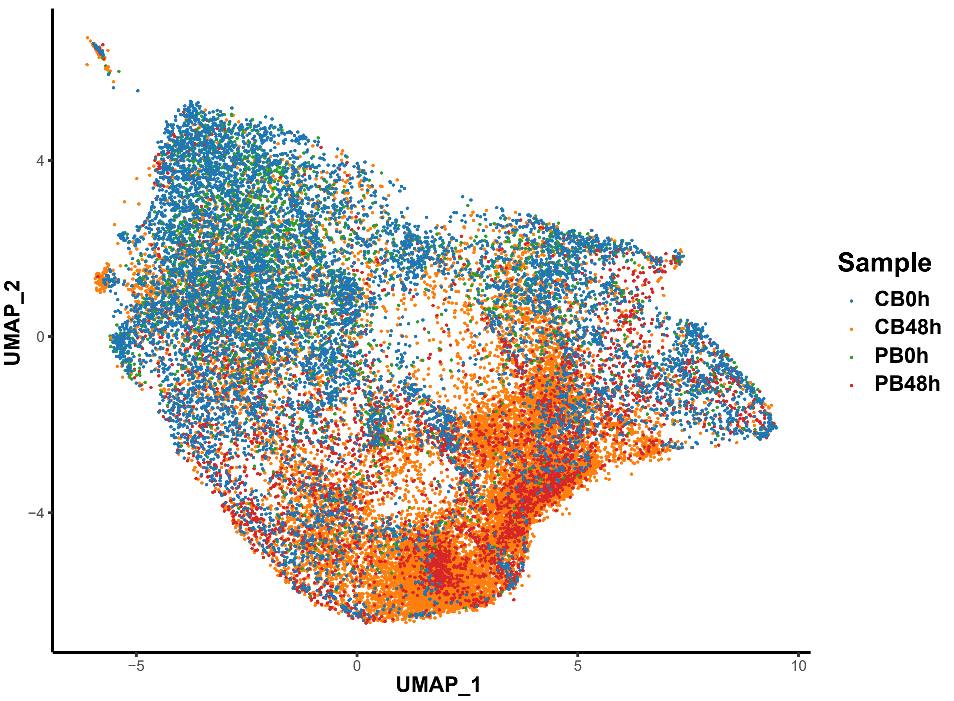

B

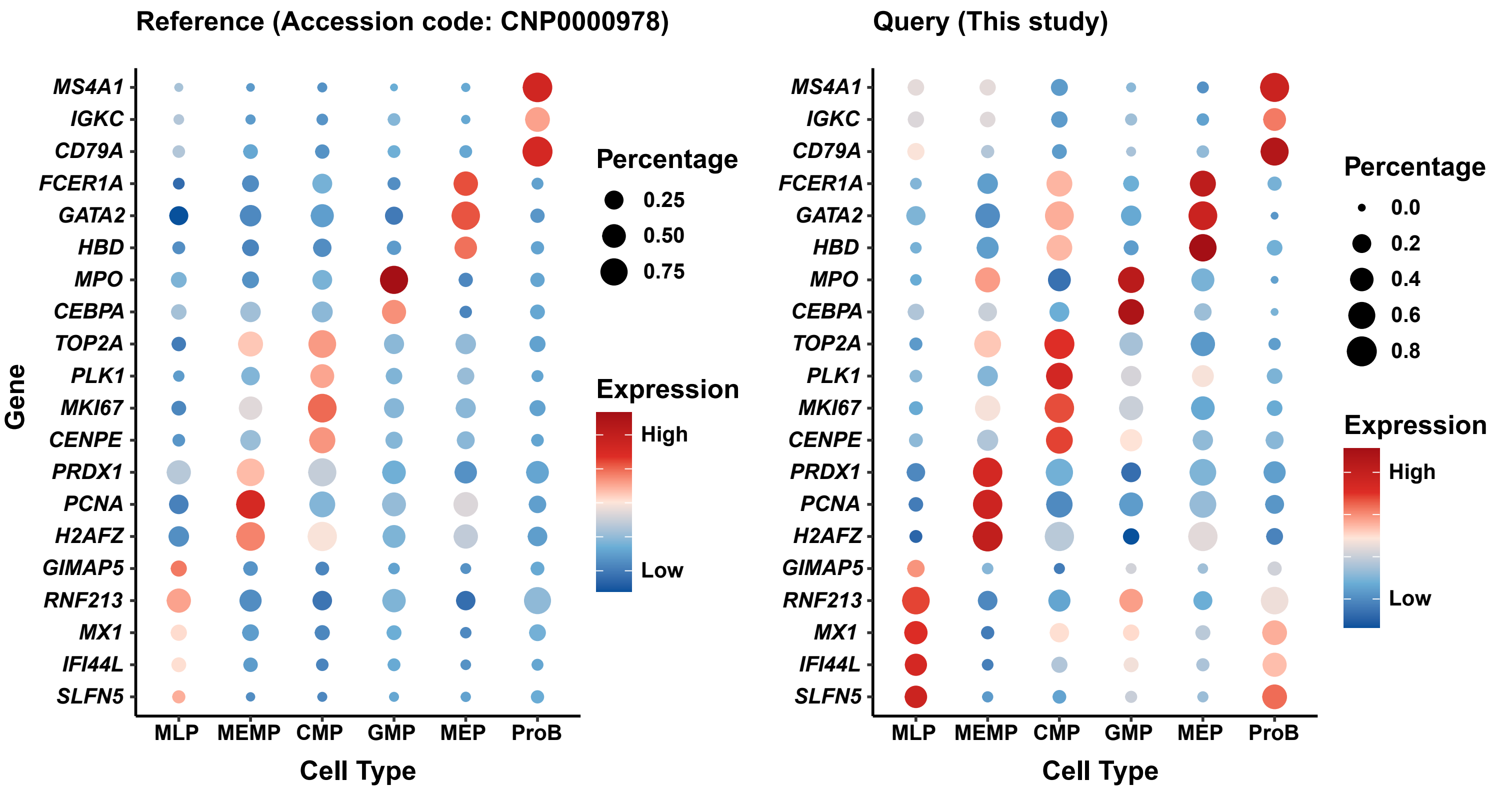

Figure S1. Samples information and the expression of cell type-specific genes in reference and query data.

(A) UMAP embedding projection of 32,303 single-cell transcriptomes (query data and reference data). Cell clusters were colored based on their sample information(see details in Methods).

(B) Dot plots illustrate the distribution of expression levels of cell-type-specific marker genes across all 6 cell types in reference data and query data (see details in Methods).
