# Supplementary material for: A novel computational method enables RNA editome profiling during human hematopoiesis from scRNA-seq data
Source: Sci Rep. 2023 Jun 26;13:10335. doi: 10.1038/s41598-023-37325-4 (PMC10293275; doi:10.1038/s41598-023-37325-4)
Supplement: Supplementary file 2 — Supplementary Legends. [file 41598_2023_37325_MOESM2_ESM.pdf]

Figure S2.The genomic elements distribution of RNA editing.
(A) Box plot showing the distance from each edited site to the nearest neighbor. **(B)** Pie charts show the proportion of editing sites within and outside ALUs in each HSPC population. **(C)** Pie charts show the distribution of editing sites across different genomic elements in each HSPC population.
