# Supplementary figures and images for: A novel computational method enables RNA editome profiling during human hematopoiesis from scRNA-seq data
Source: Sci Rep. 2023 Jun 26;13:10335. doi: 10.1038/s41598-023-37325-4 (PMC10293275; doi:10.1038/s41598-023-37325-4)

Figure S2

A

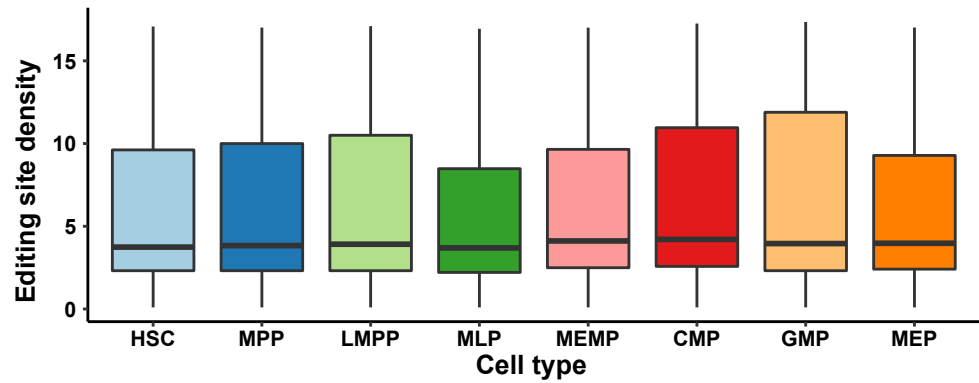

B

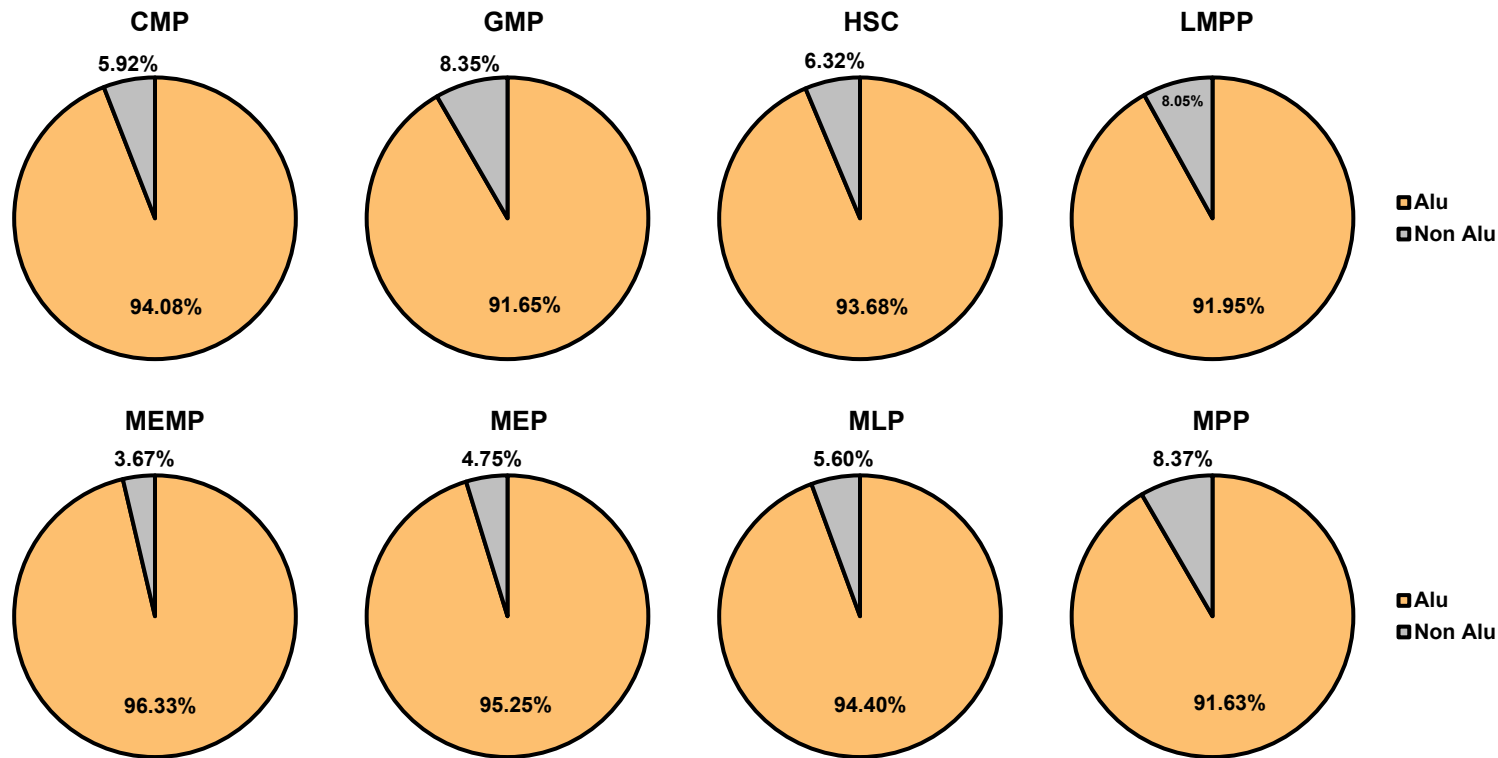

C

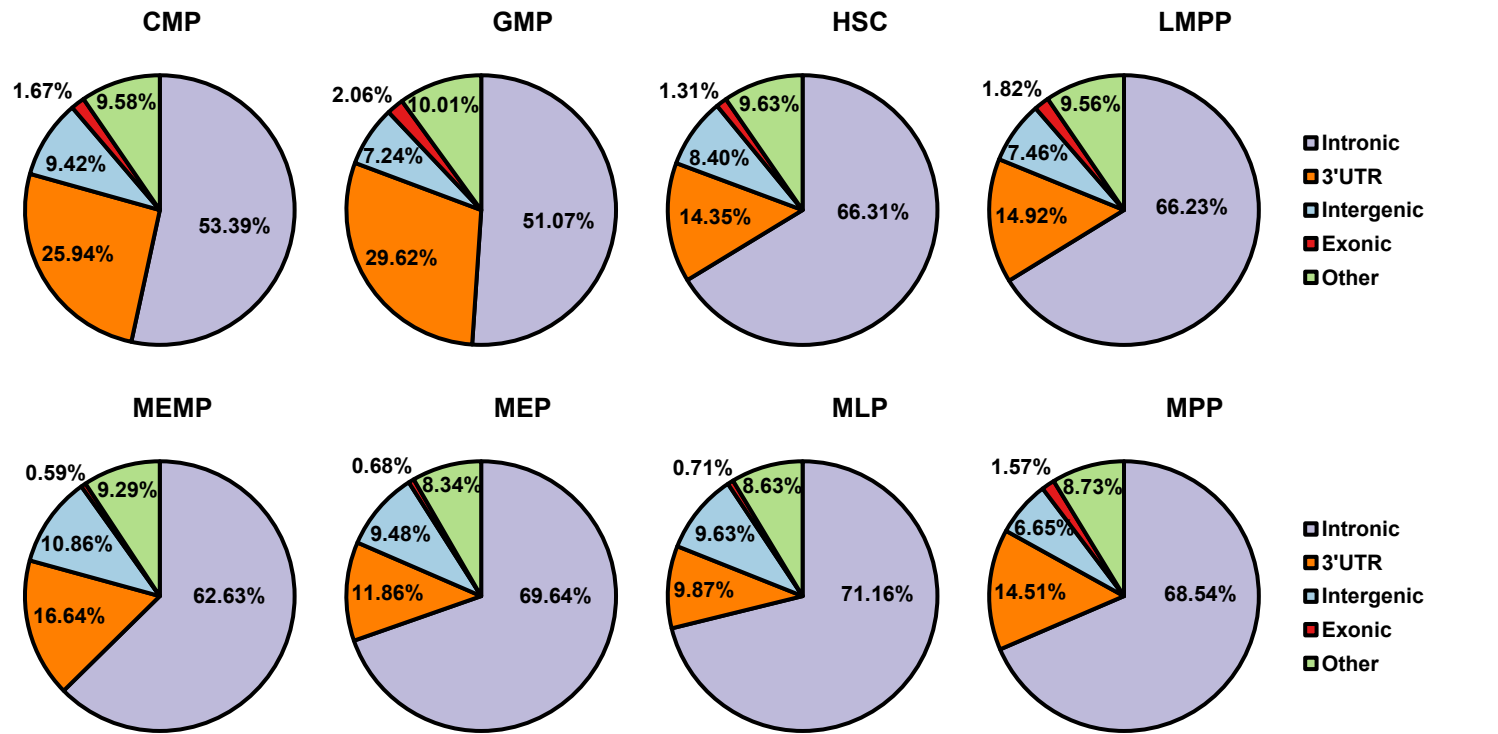

Supplement: Supplementary file 3 — Supplementary Figure S2. [file 41598_2023_37325_MOESM3_ESM.pdf]
