# Supplementary material for: A novel computational method enables RNA editome profiling during human hematopoiesis from scRNA-seq data
Source: Sci Rep. 2023 Jun 26;13:10335. doi: 10.1038/s41598-023-37325-4 (PMC10293275; doi:10.1038/s41598-023-37325-4)
Supplement: Supplementary file 4 — Supplementary Figure S3. [file 41598_2023_37325_MOESM4_ESM.pdf]

Figure S3

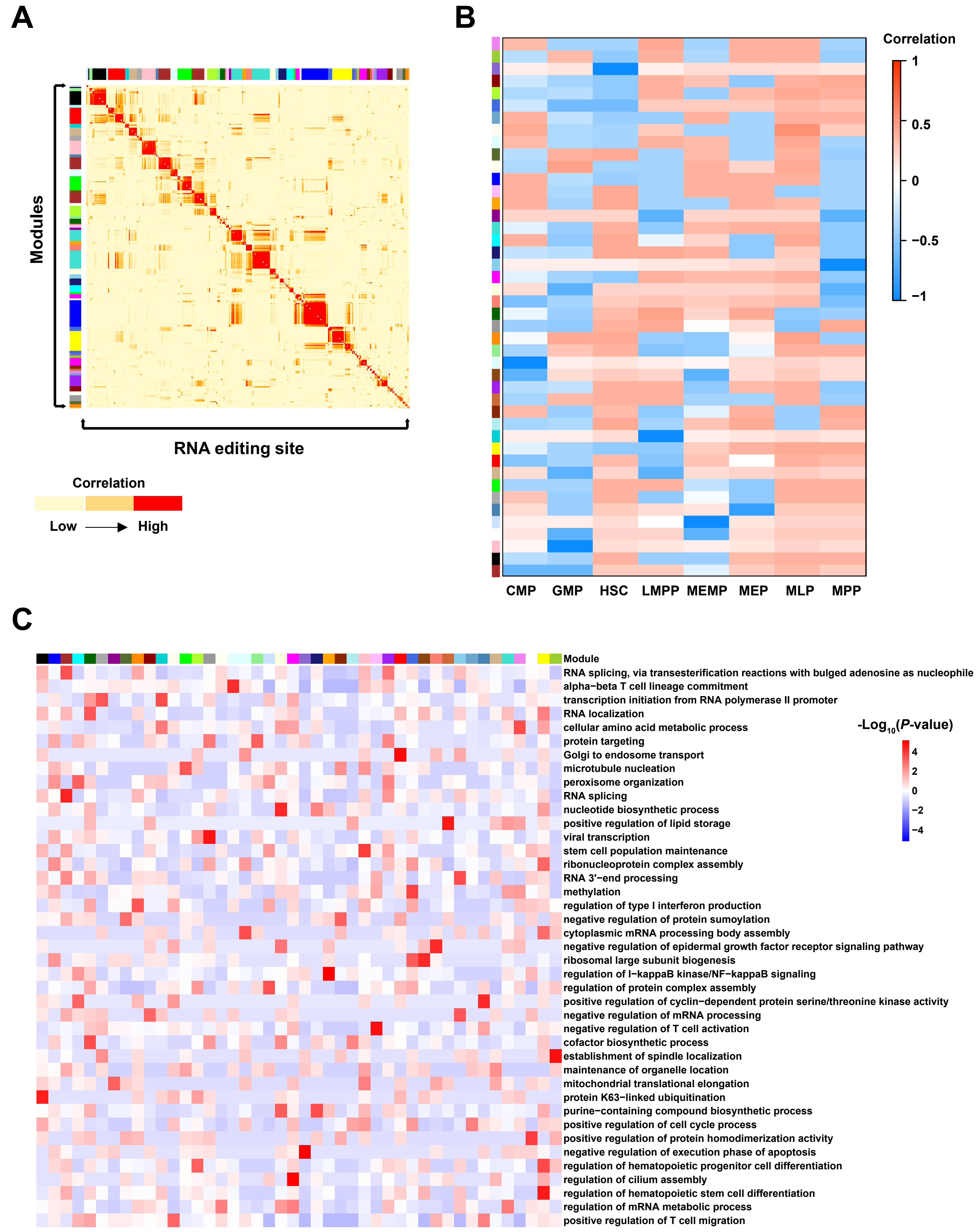

Figure S3. Co-editing modules of RNA editing and functions.
(A) A heatmap showing 44 co-editing modules across 8 HSPC populations. The color (from yellow to red) represents the correlation (from low to high). **(B)** A heatmap showing the correlation between cell types and modules. **(C)** A heatmap showing the most significant enriched GO pathways in each module.
