# Supplementary material for: A novel computational method enables RNA editome profiling during human hematopoiesis from scRNA-seq data
Source: Sci Rep. 2023 Jun 26;13:10335. doi: 10.1038/s41598-023-37325-4 (PMC10293275; doi:10.1038/s41598-023-37325-4)
Supplement: Supplementary file 5 — Supplementary Figure S4. [file 41598_2023_37325_MOESM5_ESM.pdf]

Figure S4

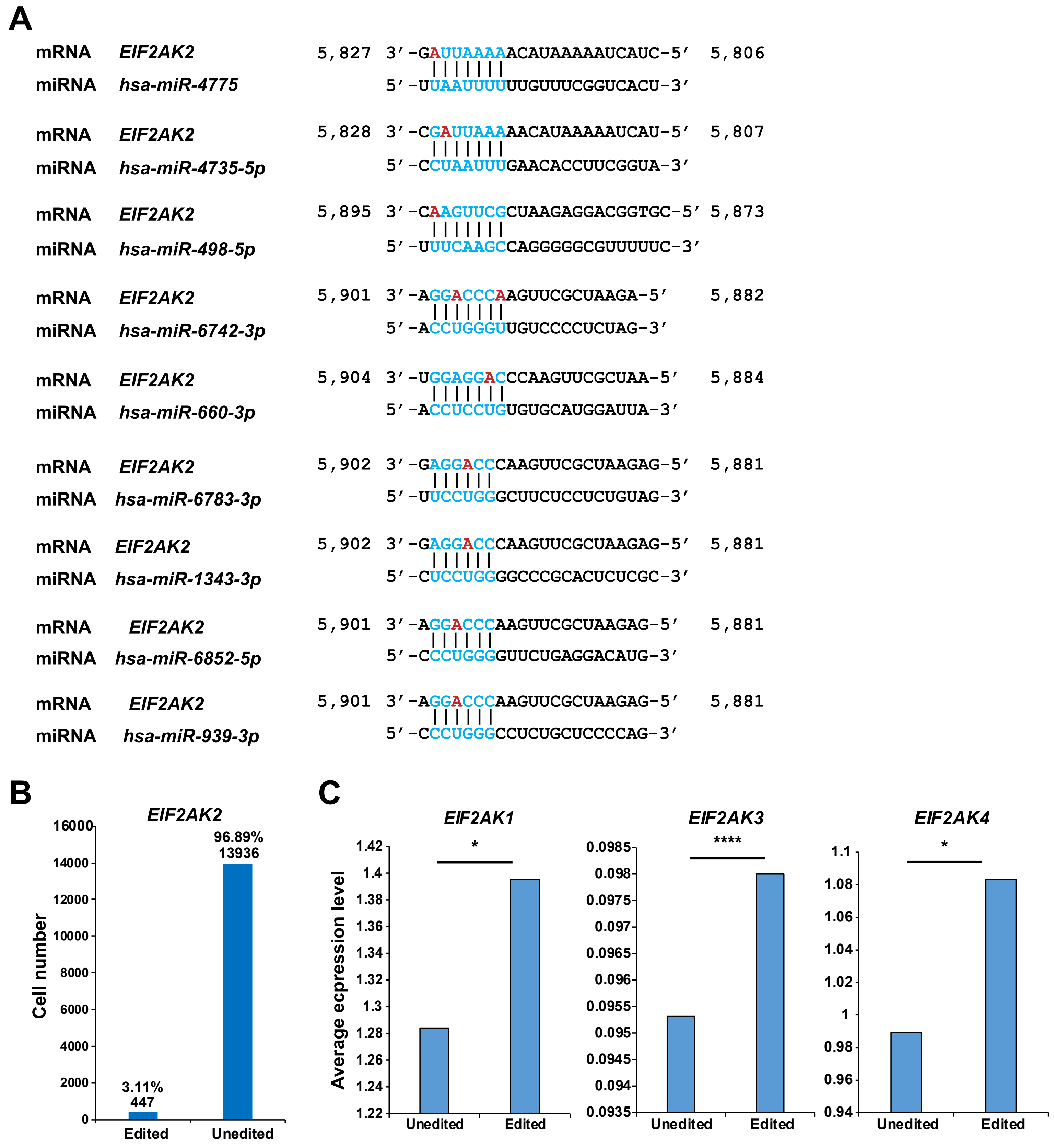

Figure S4. The RNA editing sites located in *EIF2AK2* influence miRNA-target binding.
(A) Predicted site in the *EIF2AK2* 3'UTR mRNA (red: nucleotides edited). **(B)** Bar plot showing the proportion of cells without and with EIF2AK2 editing sites. Only the cells expressing *EIF2AK2* were calculated. **(C)** Bar plots showing the average expression level of *EIF2AK1*, *EIF2AK3*, *EIF2AK4* in cells without and with *EIF2AK2* editing sites. *P*-value was calculated by Wilcoxon Rank Sum test. ****P < 0.0001, *P < 0.05
