# Supplementary material for: A novel computational method enables RNA editome profiling during human hematopoiesis from scRNA-seq data
Source: Sci Rep. 2023 Jun 26;13:10335. doi: 10.1038/s41598-023-37325-4 (PMC10293275; doi:10.1038/s41598-023-37325-4)
Supplement: Supplementary file 6 — Supplementary Figure S5. [file 41598_2023_37325_MOESM6_ESM.pdf]

Figure S5

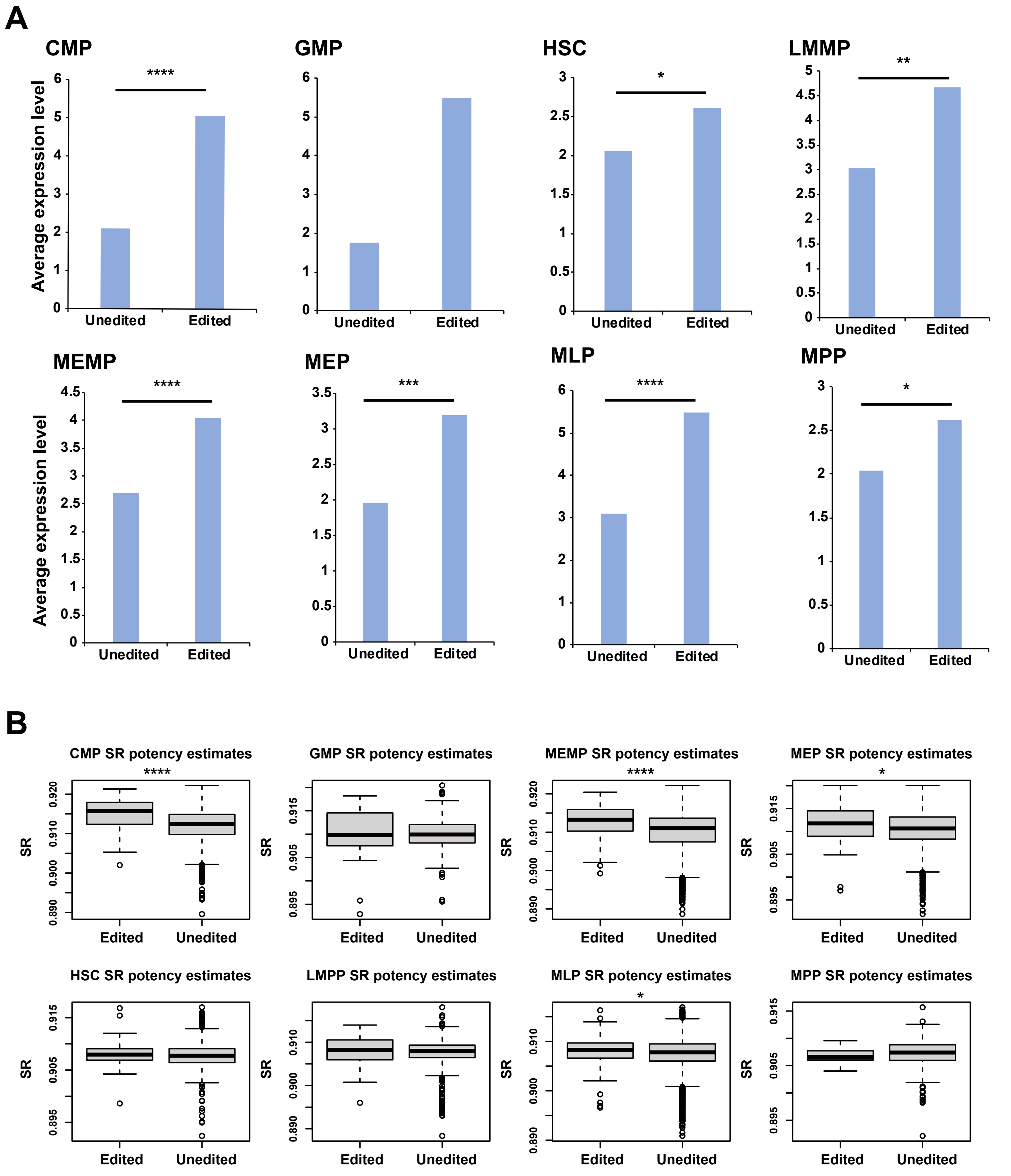

Figure S5. The 8 HSPC populaitons with editing sites may show high differentiation potential and promote the expression level of *EIF2AK2*.
(A) The bar plots show the average expression of *EIF2AK2* in 8 HSPCs of edited and unedited cell groups. ****P < 0.0001, *P < 0.05. **(B)** The boxplot shows the level of differentiation potential in 8 cell types of edited versus unedited cell populations. ****P < 0.0001, *P < 0.05. P-value was calculated by T-Test.
No * means the difference is not significant.
