# Supplementary material for: A novel computational method enables RNA editome profiling during human hematopoiesis from scRNA-seq data
Source: Sci Rep. 2023 Jun 26;13:10335. doi: 10.1038/s41598-023-37325-4 (PMC10293275; doi:10.1038/s41598-023-37325-4)
Supplement: Supplementary file 7 — Supplementary Tables. [file 41598_2023_37325_MOESM7_ESM.docx]

**SUPPLEMENTAL TABLE**

**Supplemental Table 1. The sample information.**

| Sample Source | *In vitro* culture time | Sample number |
| --- | --- | --- |
| CB | 0h | 6 |
| CB | 48h | 6 |
| mPB | 0h | 3 |
| mPB | 48h | 3 |

**Supplemental Table 2. The cell number of HSPC populations.**

| **Cell Type** | **Total cell number** |
| --- | --- |
| CMP | 2,778 |
| GMP | 974 |
| HSC | 1,599 |
| LMPP | 1,801 |
| MEMP | 13,609 |
| MEP | 5,278 |
| MLP | 4,910 |
| MPP | 1,245 |
| ProB | 109 |
